# Phytochemical Screening and Antimicrobial Activity of Methanolic Extract of *Cymbopogon schoenanthus* (L.) (azkhar) Collected from Afif City, Saudi Arabia

**DOI:** 10.3390/life13071451

**Published:** 2023-06-27

**Authors:** Leila M. Mokhtar, Inaam A. Salim, Setah N. Alotaibi, Eman A. Awaji, Maha M. Alotaibi, Ayidah O. Doman

**Affiliations:** 1 Department of Natural and Applied Sciences, College of Science and Human Studies, Shaqra University, P.O. Box 33, Shaqra 11961, Saudi Arabia; m.alaotibi@su.edu.sa; 2 Department of Chemistry, ALNairyah University College, University of Hafr ALBatin, Hafar Al Batin 39524, Saudi Arabia; iasalim@uhb.edu.sa (I.A.S.); emanawaji@uhb.edu.sa (E.A.A.); aodoman@uhb.edu.sa (A.O.D.); 3 Department of Chemistry, Faculty of Science, University of Kordofan, Al-Ubayyid 51111, Sudan; 4 Department of Home Ecnomics, College of Science and Human Studies, Shaqra University, Shaqra 11961, Saudi Arabia

**Keywords:** *Cymbopogon schoenanthus* (L.), methanolic extract, phytochemical, FTIR and NMR, antibacterial

## Abstract

In Saudi Arabia, *Cymbopogon schoenanthus* (L.) has been traditionally used to treat a variety of diseases. This study aimed to investigate the crude methanolic extract of *Cymbopogon schoenanthus* (L.) phytochemical, chemical composition, and antibacterial activity. Phytochemical analysis revealed the presence of tannins, poly-tannins, steroids, alkaloids, essential oils, terpenoids, and flavonoids. The presence of functional groups such as -COOH, -OH, -C=O, and CH_2_ was revealed via FTIR spectroscopy. ^13^C and ^1^H NMR (nuclear magnetic resonance) were used to determine the types and number of protons (hydrogen atoms) and their electronic states. Using an agar well diffusion assay, methanolic extract of *Cymbopogon schoenanthus* (L.) inhibited the growth of some foodborne pathogenic bacteria in zones ranging from 8 to 25 mm in diameter. The minimum inhibitory concentration (MIC) for *Staphylococcus aureus* was 12.5 mg/mL, whereas it was 25 mg/mL for *Bacillus cereus*, *Klebsiella pneumoniae*, and *Escherichia coli*. The time–kill assay revealed a sharp decline in *Staphylococcus aureus* and *Klebsiella pneumonia* after 2 h at a concentration of 150 mg/mL, while *Bacillus cereus* and *Escherichia coli* showed a gradual decline with constant concentrations of 75 to 150 mg/mL. The minimum bactericide concentration (MBC) value for *Bacillus cereus*, *Staphylococcus aureus*, and *Escherichia coli* was 50 mg/mL, while it was 25 mg/mL for *Klebsiella pneumoniae*. In conclusion, our study revealed that *Cymbopogon schoenanthus* (L.) methanolic extract has a significant antibacterial effect, suggesting that it could be used to treat various foodborne pathogens.

## 1. Introduction

Medicinal plants protect humans from various diseases [1]. Ancient civilizations’ knowledge of plant properties has been passed down from generation to generation [2]. To date, almost all cultures have used plants or plant-derived substances to prevent and treat disease. The use of these substances to combat microorganisms is on the rise, partially because the overuse of traditional antibiotics has allowed microbial strains to develop resistance to chemical antibiotics [3,4]. Medicinal plants were used for centuries before the advent of orthodox medicine. Leaves, flowers, stems, roots, seeds, fruit, and bark are all constituents of herbal medicines [5]. These medicinal plants are potential sources of drugs, containing secondary metabolites such as glycosides, flavonoids, alkaloids, tannins, steroids, and phenolic compounds steroids [6]. These chemical components could serve as newer leads for modern drug design [7]. The effects of chemical components in plants on the human body are identical to those of chemical compounds in conventional drugs, so herbal medicines do not differ significantly in functionality compared to conventional treatments [8]. The increasing incidence of drug-resistant pathogens has attracted the attention of the pharmaceutical and scientific communities to the potential antimicrobial activity of plant-derived substances, an untapped source of antimicrobial chemo types, which are used in traditional medicine in different countries [9,10]. Plants’ medicinal potential (antimicrobial, antioxidant, anticarcinogenic, antimalarial, immunomodulatory, etc.) has been linked to the presence of biologically active components. These biologically active substances exert a specific physiological action on the human body [5]. Plants produce poisons as a defense against predators. Many of these substances are synthesized from non-protein amino acids via biosynthetic pathways identified based on the results of isotopic tracer analysis. These secondary metabolites have been used for thousands of years, both as drugs and as agents to kill animals and commit homicide [11]. Epidemiological studies have shown that eating plant-based foods high in antioxidants is beneficial to human health because it slows down many degenerative processes and can effectively lower the incidence of cancer and cardiovascular diseases [12].

The essential oil of the plant is used in aromatherapy. These oils are natural volatile, aromatic, and complex mixtures (terpenes, phenolic compounds, alcohol) which possess antimicrobial, antioxidant, and anti-inflammatory properties. In particular, essential oils exhibit pronounced antibacterial and food preservative effects [13,14]. *Cymbopogon schoenanthus* (L.) is traditionally used as a flavoring agent in Asian cuisine; it is also consumed as a refreshing beverage. Its medicinal properties were thought to be beneficial in the treatment of prostate inflammation, kidney problems, gout, fever, rheumatism, and stomach pain. Furthermore, *Cymbopogon schoenanthus* (L.) has traditionally been used as a digestive agent to treat intestinal spasms and anorexia, as well as other uses in cosmetics and pharmaceuticals [9,15,16]. Africa and tropical Asia are all home to *Cymbopogon schoenanthus* (L.). Furthermore, *Cymbopogon schoenanthus* (L.) can be found in northern and central Sudan [17].

*Cymbopogon schoenanthus* (L.) contains saponins, saponin glycosides, flavonoids, alkaloids, triterpens, balsams, cardiac glycosides, tannins, steroids, and volatile oils [18]. It is also characterized by a high mineral content, including minerals such as iron (Fe), sodium (Na), magnesium (Mg), zinc (Zn), potassium (K), phosphorus (P), and manganese (Mn) [19].

It is primarily used in traditional Saudi medicine as an anti-infectious agent in urinary tract infections, and as a diuretic to prevent the formation of kidney stones [20]. *C. schoenanthus* essential oil was effective against *Escherichia coli*, *Staphylococcus aureus*, methicillin-sensitive (MSSA) *S. aureus* (MRSA), and *Klebsiella pneumoniae*. It was *Staphylococcus saprophyticus*, against *Staphylococcus saprophyticus*, with the highest concentration reaching >150 mg/mL. Methanolic extract showed greater inhibitory effects than essential oil [21]. The methanol extracts from the root or leaf of traditional medicinal plants showed various degrees of inhibition against bacterial strains using the agar dilution method. The antibacterial activity of the methanol extracts tested was mainly effective against Gram-negative bacteria, which have a hydrophilic outer membrane owing to their lipopolysaccharide molecular structure. Thus, small hydrophilic molecules pass through the outer membrane. This outer membrane also has properties that allow it to bypass the lipophilic compounds and macromolecules and permeating the outer membrane of the micro-organisms is prerequisite condition for any solute that exhibits antibacterial activity. The methanolic extracts have limited solubility in water; it penetrates the outer membrane of Gram-negative bacteria and disturbs their cellular function, metabolism, and cellular constituents, resulting in their death [22].

The aim of this study is to evaluate the phytochemical screening and antimicrobial activity of methanolic extract of *Cymbopogon schoenanthus* (L.) (azkhar) against susceptible and resistant pathogenic bacteria to validate some of its traditionally therapeutic properties.

## 2. Materials and Methods

### 2.1. Collection of Sample and Botanical Description

Fresh plant of *Cymbopogon schoenanthus* (L.) (azkhar) collected in January 2022 from Afif, a city in central Saudi Arabia, at the coordinates 23°54′36′′ N 42°55′13′′ E. *Cymbopogon schoenanthus* (L.) is just as versatile; it is a perennial herb with erect, tufted 9 cm-long culms, glabrous culms, and 3 to 4 nodes. It has simple, alternate, linear leaves 5–7 cm long, 1 cm wide, a sheathed apex spiny exterior, and 5 cm-long inflorescence spikelets [23,24].

### 2.2. Preparation of Plant Extract

The completely fresh plant of *Cymbopogon schoenanthus* (L.) was washed with distilled water and dried for two weeks in the shade, then subsequently ground. The maceration method was used to extract the plant sample. In the maceration vessel, 50 g of the powdered plant was macerated with 500 mL of methanol solvent. The maceration process lasted one day, with stirring at regular intervals. At 45 °C, the maceration was filtered and then evaporated.

%Yield of extract = (M1 × 100)/M2

where M1 is the extract weight after solvent removal, and M2 is the dried plant powder weight [25].

$$\mathrm{The}\;\mathrm{percentage}\;\mathrm{Yield}\;\mathrm{of}\;\mathrm{extract}=\frac{4.6}{50}\times 100=9.2\%$$

The plant extract obtained was suitable for phytochemical screening and characterization [26].

### 2.3. Phytochemical Screening

Phytochemicals are the final products of plants’ primary and secondary metabolism.

Because of their biological activity, these phytochemicals are commonly regarded as research compounds, and their health effects are still being studied. Phytochemical analysis of *Cymbopogon schoenanthus* (L.) extract was then performed using the standard protocol method [27].

#### 2.3.1. Test for Tannins (Ferric Chloride Test)

Brayme’s test: 1 mL of methanolic leaf extract of *C. schoenanthus* (L.) was added to a test tube containing 0.02 M potassium ferric cyanide, followed by 1 mL of 0.02 M ferric chloride containing 0.1 M HCl. Blue–black coloration was observed [28].

#### 2.3.2. Test for Phlobatannins

About 1 mL of *Cymbopogon schoenanthus* (L.) methanolic extract was boiled with 2% aqueous HCl, and the formation of red precipitate was used to confirm the presence of phlobatannins [29].

#### 2.3.3. Test for Saponins

In a test tube, approximately 1 mL of *C. schoenanthus* methanolic extract was mixed with 5 mL of distilled water and vigorously shaken. A few drops of olive oil were added. The presence of saponins was indicated by the formation of stable foam [30].

#### 2.3.4. Test for Flavonoids

A portion of *C. schoenanthus* methanolic extract was treated with 5 mL of dilute ammonia solution, then with concentrated H_2_SO_4_. The presence of flavonoids was indicated by the extract’s yellow coloration. Standing caused the yellow color to fade away [25].

#### 2.3.5. Test for Steroids and Terpenoids

The test used 2 mL of *Cymbopogon schoenanthus* (L.) methanolic extract with 2 mL of chloroform, 3–4 mL of acetic anhydride, and 3 mL of concentrated sulfuric acid. Dark brown coloration indicated the presence of terpenoids, and a dark blue color indicated the presence of a steroid [31].

#### 2.3.6. Test for Alkaloids

Three drops of Wagner’s reagent mixed with a methanolic extract of *Cymbopogon schoenanthus* (L.) were tested. A reddish-brown precipitate indicated the presence of alkaloids [29].

#### 2.3.7. Test for Quinones

Alcoholic KOH test: When alcoholic KOH was added to methanolic extract of *Cymbopogon schoenanthus* (L.), a red to blue color appeared, indicating a positive reaction to quinines [29].

#### 2.3.8. Test for Coumarins

In this test, 4 mL of 10% NaOH was added to 3 mL of the methanolic extract of *Cymbopogon schoenanthus* (L.). Formation of a yellow color indicated the presence of coumarin [30].

### 2.4. Chemical Composition of the Methanolic Extract of Cymbopogon schoenanthus (L.)

#### 2.4.1. FTIR (Fourier Transform Infrared Spectroscopy)

The FT-IR spectra were obtained from PerkinElmer Spectrum Version 10.03.08 using the KBr disc method (v*_max_* in cm^−1^) for sample preparation. The methanolic extract of *Cymbopogon schoenanthus* (L.) was combined with potassium bromide (KBr) (1:10) and pelletized using a hydraulic press/IR press. The pellet was then inserted into the sample slit, and the transmittance was measured [32].

#### 2.4.2. Nuclear Magnetic Resonance (^1^H, ^13^C NMR)

The NMR spectra were acquired with a Bruker Avance II 400 MHz spectrometer in deuterated chloroform (CDCl_3_) or deuterated dimethylsulfoxide (DMSO-d6) as the solvents, and the chemical shifts (δ) were measured in parts per million (ppm) downfield from tetramethylsilane (Me_4_Si) as the internal standard. The coupling constant (J) was expressed in hertz. The spin multiplicities were classified as singlet (s), broad singlet (brs), doublet (d), double doublet (dd), triplet (t), quintet (quint), doublet of triplets (dt), and triplet of double doublet (ddd) [32,33].

### 2.5. Antimicrobial Assays

#### 2.5.1. Tested Microorganisms

The American Type Culture Collection standard reference strains *Staphylococcus aureus* ATCC 29737, *Klebsiella pneumoniae* ATCC 27729, *Escherichia coli* ATCC 10537, and *Bacillus cereus* ATCC 14579 (Microbiologics Inc., St. Cloud, MN, USA) were used throughout the study.

#### 2.5.2. Agar Well Diffusion Method

The agar diffusion method was used to test the antimicrobial activity of *Cymbopogon schoenanthus* (L.) (azkhar) methanolic extract against the above-mentioned foodborne and pathogenic bacteria. One hundred microliters of each active bacterial strain grown for 24 h at 37 °C on brain Heart Infusion agar (Oxoid CM 1136, Oxoid Limited, Basingstoke, Hampshire, UK) were spread on the surface Muller Hinton agar plates (Oxoid CM 0337). The methanolic extract of *Cymbopogon schoenanthus* (L.) (azkhar) was further diluted in methanol to a final concentration of 200 mg/mL and refrigerated overnight [34]. Then, five holes were punched in the agar with a sterile cork borer with a diameter of 6 mm; four holes contained 100 μL of *Cymbopogon schoenanthus* (L.) extract in different concentrations (50, 100, 150, and 200 μg/mL, respectively, while hole number five contained 100 μL methanol as a control hole. The agar plates were then incubated at 37 °C for 24 h. The zone of inhibition of *Cymbopogon schoenanthus* (L.) (azkhar) methanolic extract (mm) was measured against different bacterial strains [35].

#### 2.5.3. Minimum Inhibitory Concentration Assay (MIC)

According to the method of Jensen et al. (2020) [36], a fresh stock solution of *Cymbopogon schoenanthus* (L.) (azkhar) methanolic extract was dissolved in methanol (99.8%) with a final concentration of 200 mg/mL for each bacterium and then diluted twofold to 50, 25, 12.5, 6.25, 3.125, and 1.562 mg/mL. In a 96-well format, the MIC for various concentrations of *Cymbopogon schoenanthus* (L.) (azkhar) methanolic extract was determined. Overnight cultures *of Staphylococcus aureus* ATCC 29737, *Bacillus cereus* ATCC 14579, *Klebsiella pneumonia* ATCC 27729, and *Escherichia coli* ATCC 10537 were diluted in nutrient broth No. 2 (Oxoid, CM0067) to 0.5 McFarland turbidity (corresponding to 106 CFU mL^-1^) [37]. In wells containing standard twofold dilutions of *Cymbopogon schoenanthus* (L.) (azkhar) methanolic extract in a final concentration of μg/mL, the bacterial suspensions were adjusted to 5 × 10^6^ CFU mL^−1^ in Mueller–Hinton (MH) broth (Oxoid, CM0405). The plates were incubated at 37 °C for 18–24 h with shaking (300 rpm). All experiments were carried out in triplicate. The MIC was defined as the concentration of *Cymbopogon schoenanthus* (L.) (azkhar) methanolic extract dissolved in methanol that inhibited the growth of tested bacteria at 600 nm [38].

#### 2.5.4. Minimum Bactericidal Concentrations (MBC)

The MBC of *Cymbopogon schoenanthus* (L.) (azkhar) methanolic extract was performed on *Staphylococcus aureus* ATCC 29737, *Bacillus cereus* ATCC 14579, *Klebsiella pneumonia* ATCC, and *Esherishia coli* ATCC 10537 in comparison to each microbe’s positive control. Next, 5 L of the corresponding inhibitory concentration and the immediately higher concentrations (MIC2 and MIC4) was subcultured on Mueller–Hinton agar Petri dishes for this purpose (Oxoid, CM0337). The MBC was determined and defined after 24 h of incubation as the lowest concentration that inhibited visible growth of the subculture [39,40].

#### 2.5.5. Time–Kill Curves

MRSA and *Staphylococcus aureus* ATCC 29737 were grown overnight at 37 °C in nutrient broth No. 2 (Oxoid, CM0067) and diluted in physiological saline (0.9% NaCl) to achieve the 0.5 McFarland turbidity, as previously described by Jensen et al. [36]. Bacterial suspensions were adjusted to 10^6^ CFU mL^−1^ in BHI containing 0, 75, and 150 mg/mL of pure *Cymbopogon schoenanthus* (L.) (azkhar) methanolic extract in a final volume of 100 mL and incubated at 37 °C with aeration (150 rpm). Cell counts were determined through tenfold serial dilution on nutrient agar (Oxoid, CM0003) every hour for the first 4 h, then 8 and 24 h later. Experiments were carried out in duplicate. The MIC of overnight cultures was determined for one of the duplicate experiments to determine susceptibility after prolonged exposure [36].

## 3. Results and Discussion

### 3.1. Phytochemical Screening and Chemical Analysis of the Methanolic Extract

**Figure 1:**
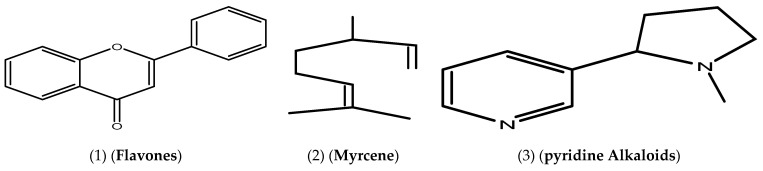
Active compounds were identified by comparing the retrieved compounds with the standard char.

**Figure 2:**
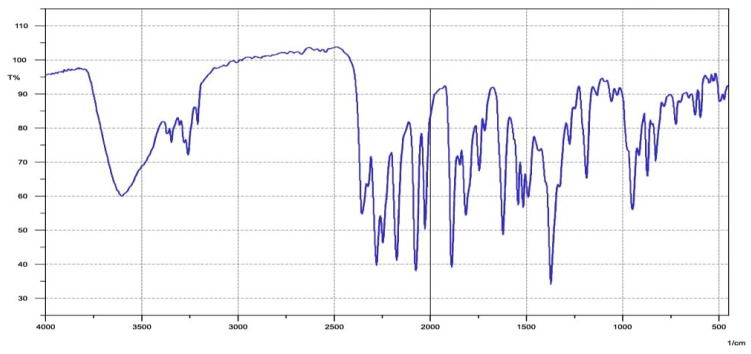
IR spectrum crude methanolic extract of *Cymbopogon schoenanthus* (L.) (azkhar).

**Figure 3:**
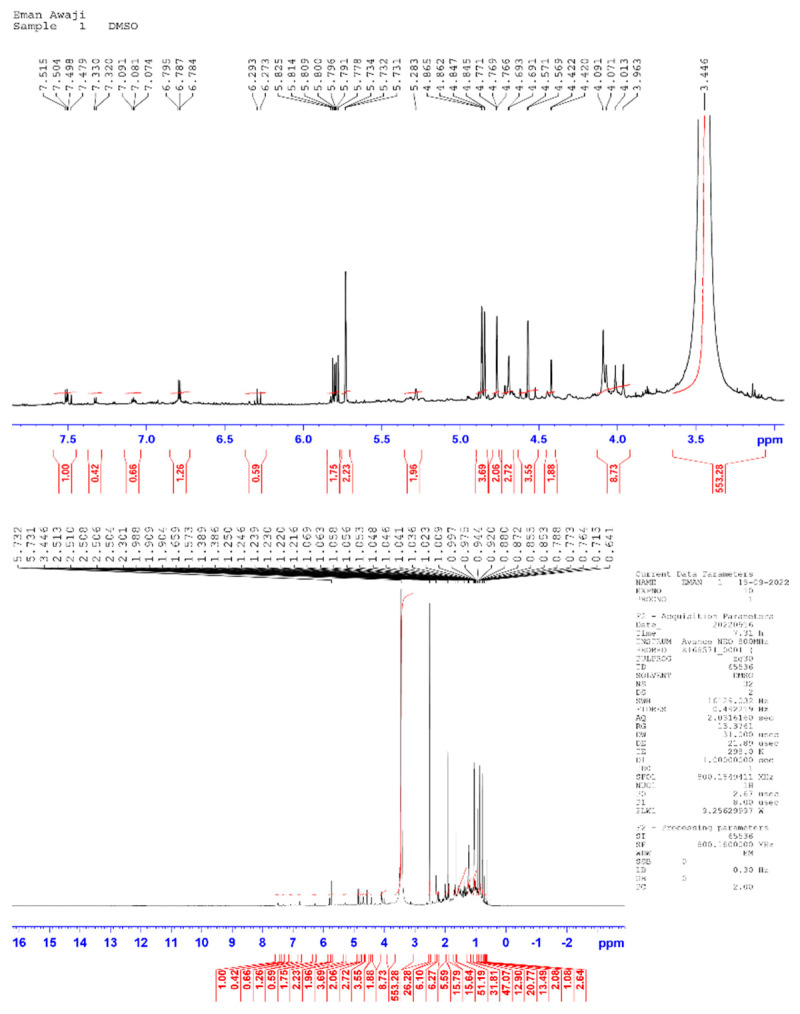
^1^H NMR spectrum of crude methanolic extract of *Cymbopogon schoenanthus* (L.) (azkhar).

**Figure 4:**
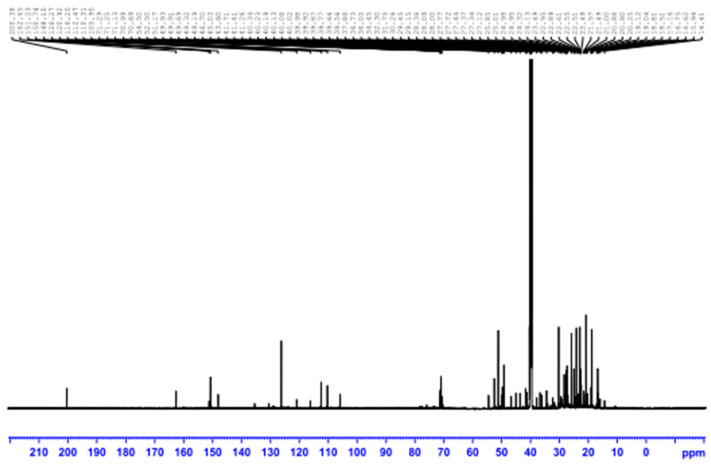
^13^C NMR spectrum crude methanolic extract of *Cymbopogon schoenanthus* (L.) (azkhar).

As shown in Table 1, biologically active substances, including tannins, polytannins, steroids, terpenoids, qumarins, alkaloids, and flavonoids, were found in *Cymbopogon schoenanthus* (L.) methanolic extract, but saponins and quinones were not, as reported by Khatun et al. [41]. Our findings are consistent with those reported by EL-Kamali and AL-Amir [42] and Mustafa et al. [43]. The vast amount of information gathered from the ethno-pharmacological applications of *Cymbopogon schoenanthus* (L.) demanded that its chemical constituents be investigated. These investigations resulted in the isolation of alkaloids, volatile and non-volatile terpenoids, flavonoids, and tannins from all parts of these plants [44]. The presence of various functional groups such as -OH, -COOH, -CH_2_, and C=O was revealed by FTIR spectroscopy from the IR absorption bands in the high wavelength region at 3456 cm^−1^ and 2939 cm^−1^, and the active compounds were identified by comparing the retrieved compounds with the standard char (Table 2 and Figure 1). ^1^H and ^13^C NMR were used to determine the number of protons present and their electronic states in the various compounds (Table 3, Figure 2, Figure 3 and Figure 4). The presence of monoterpenes (*compound #1*) and sesquiterpenes (*compound #2*), flavones, and alkaloids (*compound #3*) was detected via chemical analysis.

### 3.2. Antibacterial Activities of Cymbopogon schoenanthus (L.) (azkhar)

#### 3.2.1. Agar Diffusion Method

As illustrated in Table 4, with the increasing the concentration of *Cymbopogon schoenanthus* (L.) and methanolic extract 50, 100, 150, and 200 mg/mL, directly in proportion to the increasing of the zone of inhibition against different tested bacteria strains. These findings demonstrate that the phenolic and flavonoid compounds found in *Cymbopogon schoenanthus* (L.) exhibit biological activity against all Gram-positive and Gram-negative bacterial strains tested. *Bacillus cereus* had the greatest inhibitory effect and was 25 mm in diameter, followed by *Staphylococcus aureus* (22 mm), *Klebsiella pneumonia* (21 mm), and *Escherichia coli* (16 mm), at 200 mg/mL, respectively. Hashim et al. [45] found that the essential oil of *Cymbopogon schoenanthus* (L.) exhibited antibacterial activity against 50% of the pathogenic bacteria tested in their study. Bacterial pathogens were tested. El-Kamali et al. [46] discovered that essential oil of *Cymbopogon schoenanthus* (L.) nervatus exhibited antibacterial activity against some Gram-positive and Gram-negative bacteria such as *Staphylococcus aureus*, *Bacillus subtilis*, *Escherichia coli*, *Pseudomonas aeruginosa*, *Salmonella paratyphi* A, *Salmonella paratyphi B*, *Shigella dysenteriae*, *Shigella flexneri*, *Shigella boydii*, *P. mirabilis*, *and Klebsiella pneumoniae*. They discovered that *S. dysenteriae* and *Klebsiella pneumonia* had the greatest inhibitory effect. *Cymbopogon schoenanthus* (L.) dissolved in ethanol or chloroform exhibited antibacterial activity against *Staphylococcus aureus*. According to some authors, Gram-negative bacteria are more resistant to the essential oil of *Cymbopogon schoenanthus* (L.) (azkhar) than Gram-positive bacteria due to the structure of the outer layer of the cell membrane, which consists of dense lipopolysaccharide molecules and perivascular enzymes in Gram-negative bacteria [47,48]. Therefore, antimicrobial compounds can easily disintegrate the cell walls and cytoplasmic membranes of some Gram-positive bacteria and release their cytoplasmic contents, ultimately leading to their death [49,50]. According to Mohamed et al. [43], the highest level of antibacterial activity was demonstrated by *Cymbopogon schoenanthus* (L.) ethanolic extract against all isolated bacteria *Staphylococcus aureus*, *Escherichia coli*, *Klebsiella pneumoniae*, and *Pseudomonas aeruginosa*, with inhibitory zones ranging from 24.6 to 10 mm. Additionally, Khalil et al. [51] reported that the methanol extract of *Cymbopogon schoenanthus* (L.) exhibited antibacterial activity against several Gram-positive and Gram-negative bacteria. All the extracts tested (aqueous and methanol extracts) were found to have an antiviral effect on HSV1, but no antifungal effect was found. They also stated that the inhibition zones ranged from 22 to 19.3 mm. Gasal et al. [52] noted similar results when using water extracts of *Cymbopogon schoenanthus* (L.) against ten Gram-positive and Gram-negative bacteria isolates.

#### 3.2.2. Minimum Inhibitory Concentration

Table 5 shows that the MIC of *Cymbopogon schoenanthus* (L.) (azkhar) methanolic extract for *Bacillus cereus* ATCC 14579 was 12.5 mg/mL, while that of *Staphylococcus aureus* ATCC 29737, *Klebsiella pneumonia* ATCC 27729, and *Escherichia coli* ATCC 10537 was 25 mg/mL. According to Al Yahya et al., 1983 [19]. Additionally, as described by Lahlou, 2004 [53], the MICs of *Cymbopogon schoenanthus* (L.) and *C. nervatus* essential oil extracts for *S. aureus* were higher than reported by Hashim et al., 2017 [45]. This disparity could be due to the method of essential oil extraction. It is not uncommon to note significant differences in data for the same plant species. Many factors, including the method of essential oil extraction, climatic, seasonal, and geographical conditions, and harvest time, may contribute to this variation. To reduce these differences, standardized extraction methods and conditions must be limited and precise. Abdoul-Latif et al., 2022 [54], discovered that the essential oil of *Cymbopogon schoenanthus* (L.) has antibacterial activity against only four bacteria: *Escherichia coli*, *Micrococcus lutes*, *Klebsiella pneumonia*, and *Shigella sonnei*. This was supported by Aly et al. (2017) [55].

#### 3.2.3. Minimum Bactericide Concentration (MBC) and Time–Kill Curves

**Figure 5:**
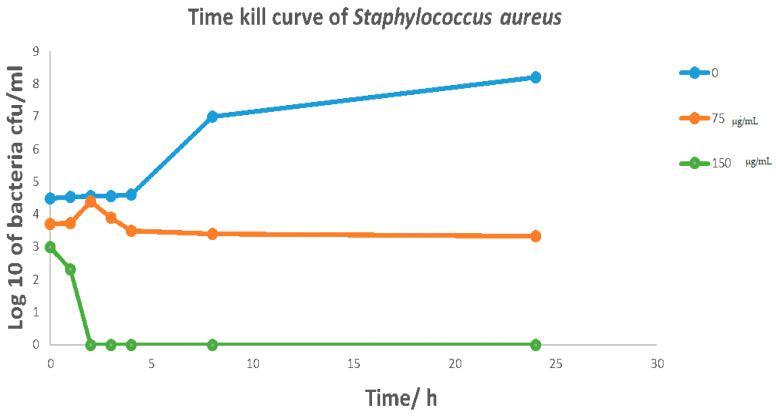
Effect of methanolic extract of *Cymbopogon schoenanthus* (L.) (azkhar) at a concentration of 0, 75, 150 mg/mL, on the time–kill curves of *Staphylococcus aureus* ATCC 29737 (Overnight cultures = 10^6^ CFU mL^−1^). Viability of bacteria was evaluated after exposure to methanolic extract by determining CFU mL^−1^ every hour for the first 0–4 h and then after 8 and 24 h.

**Figure 6:**
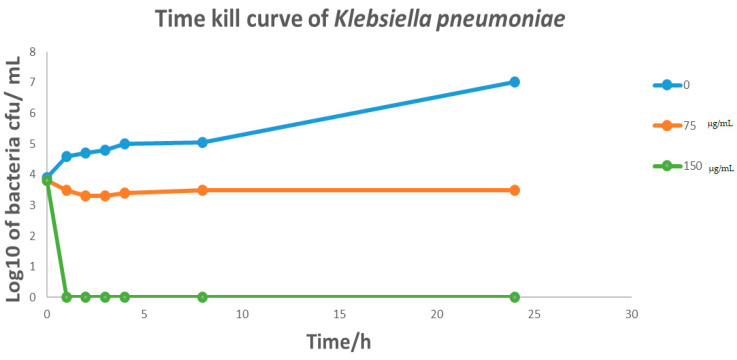
Effect of methanolic extract of *Cymbopogon schoenanthus* (L.) (azkhar) at a concentration of 0, 75, 150 mg/mL, on the time–kill curves of *Klebsiella pneumonia* ATCC 27729 (Overnight cultures = 10^6^ CFU mL^−1^). Viability of bacteria was evaluated after exposure to methanolic extract by determining CFU mL^−1^ every hour for the first 0–4 h and then after 8 and 24 h.

**Figure 7:**
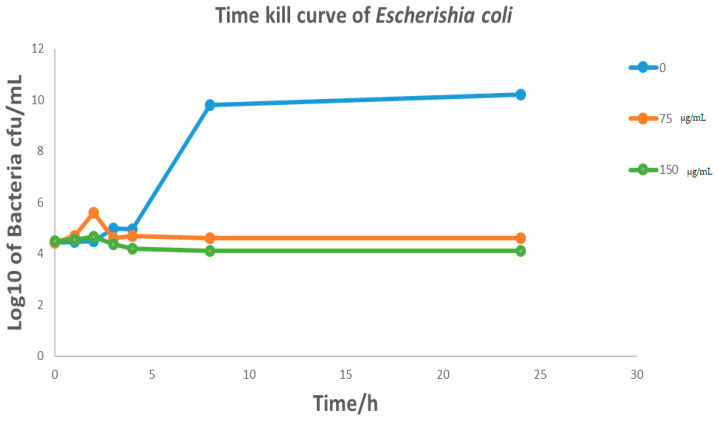
Effect of methanolic extract of *Cymbopogon schoenanthus* (L.) (azkhar) at a concentration of 0, 75, 150 mg/mL, on the time–kill curves of *Escherichia coli* ATCC 10537 (Overnight cultures = 10^6^ CFU mL^−1^). Viability of bacteria was evaluated after exposure to methanolic extract by determining CFU mL^−1^ every hour for the first 0–4 h and then after 8 and 24 h.

**Figure 8:**
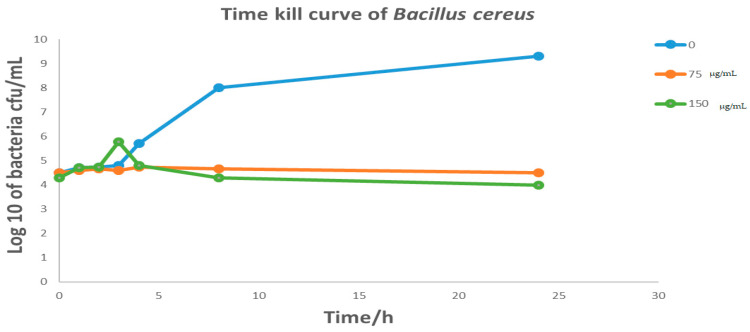
Effect of methanolic extract of *Cymbopogon schoenanthus* (L.) (azkhar) at a concentration of 0, 75, 150 mg/mL, on the time–kill curves of *Bacillus cereus* ATCC 14579 (Overnight cultures= 10^6^ CFU mL^−1^). Viability of bacteria was evaluated after exposure to methanolic extract by determining CFU mL^−1^ every hour for the first 0–4 h and then after 8 and 24 h.

*Cymbopogon schoenanthus* (L.) (azkhar) MBC was 50 mg/mL for *Bacillus cereus*, *Staphylococcus aureus*, and *Escherichia coli*, and 12.5 mg/mL for *Klebsiella pneumonia*. As a result, *Cymbopogon schoenanthus* (L.) (azkhar) is thought to have a bactericide effect against all Gram-positive and Gram-negative bacteria tested, as shown in Table 6. The time–kill assay revealed a sharp time decline in *Staphylococcus aureus* at a *Cymbopogon schoenanthus* (L.) (azkhar) concentration of 150 mg/mL after 2 h of incubation, followed by a gradual reduction in growth at 75 mg/mL after 5 h. A constant state was then maintained until the end of the incubation period (Figure 5). Hashim et al. (2017) [45] reported a time-dependent decline in the case of *Staphylococcus aureus*, with a 90% reduction achieved within 8 h of exposure to *Cymbopogon schoenanthus* (L.) essential oil. The time–kill assay revealed a sharp time decline in *Klebsiella pneumonia* at a *Cymbopogon schoenanthus* (L.) (azkhar) concentration of 150 mg/mL after 1 h of incubation, followed by a gradual reduction in growth at 75 µg/mL after 1 h, after which a constant state was maintained until the end of the incubation period (Figure 6). Hashim et al. (2017) [45] reported that *Klebsiella pneumonia* was much more sensitive, with 99.95% inhibition achieved within the first two hours of contact with *Cymbopogon schoenanthus* (L.) essential oil [45]. The results agreed with our data. The time–kill assay revealed that at *Cymbopogon schoenanthus* (L.) (azkhar) concentrations of 75 and 150 mg/mL, there was a gradual decline and then a constant state for both *Bacillus cereus* and *Escherichia coli* until the end of the incubation period (Figure 7 and Figure 8). According to some authors, *Cymbopogon schoenanthus* (L.) oil has a bacteriostatic effect on bacteria [18]. Mclaughlin et al. (1998) [56] investigated the composition of plant extracts and discovered alkaloids, steroids, saponins, glycosides, and flavonoids that may have medicinal and antibacterial properties [57,58]. Finally, the effect of the methanolic extract of *Cymbopogon schoenanthus* (L.) (azkhar) on bacteria may be focused on the cell wall and cell membrane lysis and death. The effects on DNA, RNA, proteins, and polysaccharides were also examined; they were found to inhibit microbial cells [59].

## 4. Conclusions

Biologically active substances, including tannins, polytannins, steroids, terpenoids, coumarins, alkaloids, and flavonoids, were found in *Cymbopogon schoenanthus* (L.) methanolic extract, but saponins and quinones not. The extract exhibited antibacterial activities. The zone of inhibition was increased with the concentration of methanolic extracts, hindering bacterial growth. Further studies on the structure of bioactive compounds of *Cymbopogon schoenanthus* (L.) extracts with different organic solvents will be elucidated using different advantage analysis to gain a better understanding of this topic.

## Figures and Tables

**Table 1:** Phytochemical analysis of a methanolic extract of *Cymbopogon schoenanthus* (L.) (azkhar).

Phytochemicals	Result
Tannins	+++
Phlobatannins	+++
Saponins	--
Flavonoids	++
Steroid	++
Alkaloid	+++
Quinons	--
Terpenoids	+++
Qumarins	+++

+++ strong reaction intensity; ++ medium reaction intensity; -- non-detected [6] (Marka et al. 2013).

**Table 2:** Peak values, band type, and functional group for FTIR (Fourier Transform Infrared Spectroscopy) spectra of methanolic extract of *Cymbopogon schoenanthus* (L.) (azkhar).

Wavenumber (ν cm^−1^)	
Spectrum	Bond
2900–3000	O-H Phenol
3100	C-H aromatic
1580–1430	C=C aromatic
1800	C=O ketone
2950	C-H aliphatic
1600	C=C aliphatic
1150–1100	C-O-C aliphatic

**Table 3:** ^1^H, ^13^C NMR spectral data of *Compounds* (1), (2),(3).

^13^C NMR δ (ppm) in DMSO/CDCl_3_	^1^H NMR δ (ppm)in DMSO/CDCl_3_	*Compound #*
200 (C=O) 116–152 (12C-aromatic) 105,162 (C=CH)	6.80–7.50 (d,t9H-aromatic) 5.70 (s,HC=C)	(1)
36 (HC-CH_3_) 16, 18, 26 (3CH_3_) 148 (HC=CH_2_) 115 (HC=CH_2_) 125 (HC=C(CH_3_)_2_) 134 (HC=C(CH_3_)_2_) 31, 37 (H_2_C-CH_2_)	1.4 (q,HC-CH_3)_ 0.6 (d,HC-CH_3_) 5.80 (t,HC=CH_2_) 4.90 (s,HC=CH_2_) 5.40 (s,HC=C(CH_3_)_2_) 1.95, 2.3 (t, H_2_C-CH_2_) 1.60, 1.85 (s,2CH_3_)	(2)
43 (CH_3_-N) 121–127 (5C-aromatic) (3CH_2_)	1.60:1.53–2:1.77–2.30:2.50 (t,q,3CH_2_) 3.90 (t, HC-CH_2_) 7.10–7.30 (s, d, t, 4H-aromatic)	(3)

**Table 4:** Zone of inhibition (mm) of methanolic extract of *Cymbopogon schoenanthus* (50–200 mg/mL) against some Gram-positive and -negative bacteria.

	Concentrations (mg/mL)	M ± SD			
Bacterial Strains		50	100	150	200
*Staphylococcus aureus* ATCC 29737		9 ± 0.816	12 ± 1.632	18 ± 1.414	22 ± 1.633
*Klebsiella pneumonia* ATCC 27729		10 ± 1.633	14 ± 0.816	16 ± 0.816	21 ± 1.414
*Escherichia coli* ATCC 10537		8 ± 0.094	12 ± 1.4142	15 ± 0.816	16 ± 0.957
*Bacillus cereus* ATCC 14579		18 ± 0.471	20 ± 0.8164	22 ± 0.816	25 ± 0.957

Values are means of three replicates. Results were given as mean (M) ± standard deviation (SD).

**Table 5:** Minimum Inhibitory concentration of methanolic extract of *Cymbopogon schoenanthus* (L.) (azkhar).

	Bacterial Strains	*Bacillus cereus* ATCC 14579	*K. pnuemoniae* ATCC 27729	*Staphylococcus aureus* ATCC 29737	*E. coli* ATCC 10537
Concentration µg/mL		Optical Density (O.D) at 600 nm			
100		0	0	0	0
50		0	0	0	0
25		0	0.744	0.848	1.385
12.5		1.251	0.796	0.804	1.493
6.25		1.185	0.862	0.758	1.243
3.125		1.119	0.929	0.713	1.28
1.562		0.858	0.947	0.68	1.263

0 = no growth.

**Table 6:** Minimum bactericide concentration of methanolic extract of *Cymbopogon schoenanthus* (L.) (azkhar).

	Bacterial Strains	*Bacillus cereus* ATCC 14579	*Klebsiells pnuemoniae* ATCC 27729	*Staphylococcus aureus* ATCC 29737	*Esherishia coli* ATCC 10537
Concentration µg/mL					
100		-	-	-	-
50		+++	-	+++	+++
25		+++	-	+++	+++
12.5		+++	+++	+++	+++
6.25		+++	+++	+++	+++
3.125		+++	+++	+++	+++
1.175		+++	+++	+++	+++
0.75		+++	+++	+++	+++
0.25		+++	+++	+++	+++

+++ = Good growth, - = No growth.

## Data Availability

The data presented in this study are available on request from the corresponding author.
